# Prevalence of breastfeeding in a baby-friendly pediatric practice in Trieste, Italy: follow up to 36 months of age

**DOI:** 10.1186/s13006-021-00441-w

**Published:** 2021-12-14

**Authors:** Mariarosa Milinco, Adriano Cattaneo, Anna Macaluso, Paola Materassi, Nicola Di Toro, Luca Ronfani

**Affiliations:** 1 Clinical Epidemiology and Public Health Research Unit, Institute for Maternal and Child Health - IRCCS “Burlo Garofolo”, Trieste, Italy; 2 Epidemiologist, Trieste, Italy; 3 Pediatric practice, Trieste, Italy

## Abstract

**Background:**

A breastfeeding-friendly physician’s office that applies the 13 recommendations of the Academy of Breastfeeding Medicine can help increase the exclusivity and duration of breastfeeding. Having already published the results up to five months of age of this intervention in our pediatric practice, we now report on the follow up to 36 months.

**Methods:**

A cohort of 252 newborn infants was enrolled with our pediatric office in Trieste, Italy, between 1 January 2016 and 31 December 2016. The office implemented baby-friendly pediatric practices and a biological nurturing approach to the support of breastfeeding. In addition to the services offered by two pediatricians, support was provided by a peer counselor. Data on breastfeeding were collected at periodic healthy child visits up to 36 months of age. The outcome of interest for this follow up was the rate of any breastfeeding, defined as the percentage of infants and children who had received breastmilk in the previous 24 h.

**Results:**

The rates of any breastfeeding at discharge and at 1, 3 and 5 months (*n* = 252) were 95.2, 95.8, 89.3 and 86.5%, respectively. At 8, 12, 18, 24 and 36 months of age, the rates of breastfeeding were 70.6% (163/231), 59% (135/229), 35% (78/224), 24.6% (55/224) and 7.2% (16/224), respectively.

**Conclusions:**

The rates of any breastfeeding recorded in our pediatric practice up to age 36 months, are much higher than those reported elsewhere in high income countries and are likely to be associated with our baby-friendly and biological nurturing approach.

## Background

According to the Academy of Breastfeeding Medicine [1], a breastfeeding-friendly physician’s office should implement the following steps: 1. establish a written policy; 2. educate all office staff on breastfeeding support skills and implement the skills with patients; 3. comply with the International Code of Marketing of Breastmilk Substitutes; 4. know local and national breastfeeding laws; 5. promote breastfeeding in the office; 6. normalize breastfeeding; 7. consider breastfeeding when providing prenatal care; 8. help mothers initiate and continue breastfeeding; 9. bridge postpartum care to the outpatient setting; 10. encourage cross-disciplinary care; 11. educate patients; 12. educate healthcare providers; 13. collect breastfeeding data. In 2019, we published an article showing that the implementation of these steps in a pediatric practice of Trieste, Italy, together with the adoption of a biological nurturing approach [2] and the employment of a peer counsellor [3], can lead to high rates of exclusive breastfeeding up to five months of age [4]. In this short communication, we report on the follow up of the same children up to 36 months of age.

## Methods

The 252 newborn infants with gestational age greater than 30 weeks, born between 1 January 2016 and 31 December 2016 and registered with our pediatric practice, whose rates of any breastfeeding up to five months of age have already been published [4], were followed up to age 36 months. Breastfeeding data were collected at periodic child health visits scheduled at 8, 12, 18, 24 and 36 months of age. The outcome of interest for this follow up was the rate of any breastfeeding, defined as the percentage of infants and children who had received breastmilk in the previous 24 h, according to WHO standards [5].

## Results

The characteristics of the study population are described in our previous report [4]. About 30% of parents were not Italian, more than 80% of mothers had a high level of education, about two thirds were employed, 58% were 30 to 39 years of age, 72% were primiparae and 21% had delivered by cesarean section.

**Fig. 1:**
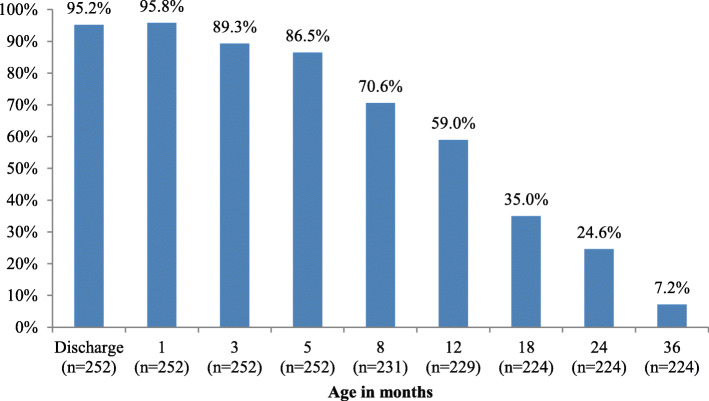
Rates of any breastfeeding between discharge and 36 months of age

Figure 1 shows the rates of any breastfeeding between discharge from the hospital up to 36 months of age. The rates of exclusive breastfeeding at discharge and at 1, 3 and 5 months were 67.9% (171/252), 71.8% (181/252), 71.8% (181/252) and 62.3% (157/252), respectively; those of any breastfeeding were 95.2% (240/252), 95.8% (241/252), 89.3% (225/252) and 86.5% (218/252). After age five months, 28 children (11%) were lost to follow up. At 8, 12, 18, 24 and 36 months of age, the rates of breastfeeding were 70.6% (163/231), 59% (135/229), 35% (78/224), 24.6% (55/224) and 7.2% (16/224), respectively.

## Discussion

This is one of the rare reports in the literature on rates of breastfeeding up to 36 months of age. In a cohort of 400 newborn infants recruited between July 2007 and July 2008 in the Friuli Venezia Giulia region, where Trieste is located, the rates of any breastfeeding were much lower: 39, 20, and 12% at 12, 18 and 24 months, respectively [6]. Much lower rates are reported also from other high-income countries. In South Australia, for example, a study on a cohort of more than 2000 mother and child dyads recruited in 2013/14 showed rates of any breastfeeding of 31.8, 12.1 and 7.5% at 12, 18 and 24 months [7]. Similar or even lower rates were reported from Canada and the USA [8, 9]. In the WHO European region, only 25 out of 45 Member States have reported breastfeeding rates at 12–15 months of age, with percentages ranging from 1 to 78%. Only 5 countries reported a higher rate than ours (59%): Uzbekistan 78%, Turkmenistan 72%, Kyrgyzstan 68%, Turkey 67% and Albania 61% [10]. In a more recent article with data from 51 high income countries, only two out of 24 had higher breastfeeding rates at 12 months than ours (Oman and Bahrain); of the five countries for which data at 24 months were available, only two (Oman and Uruguay) reported rates higher than our 24.6% [11].

In an observational study, it is impossible to prove a causal association between intervention and outcome. Yet, increases in breastfeeding rates were reported by two before-and-after studies that implemented a similar protocol for baby-friendly pediatric practices [12, 13]. In addition, there is evidence of a positive effect for two of the components of our composite intervention. First, when professional and peer support is provided, breastfeeding rates increase [14]. Second, biological nurturing and the laid-back position reduce breast problems [15, 16], and can thus promote a better establishment and continuation of breastfeeding. As secular trends are unlikely to explain our results, the most likely explanation for our high breastfeeding rates is the application of our composite intervention.

In addition to its observational design and its relying on routine data collected during child health visits, our study had three further limitations. It did not include preterm infants, who are usually exposed to different routines in pediatric practices, it reported some loss to follow up at 36 months of age, and possibly suffered from a selection bias, due to the fact that our pediatric practice may have attracted a higher proportion of mothers wishing to breastfeed longer. It is unlikely, however, that these limitations altered significantly our results.

## Conclusions

The rates of any breastfeeding up to age 36 months recorded in our pediatric practice are much higher than those reported elsewhere in high income countries and are likely to be associated with our baby-friendly and biological nurturing approach.

## Data Availability

The dataset used during the current study is available from the corresponding author on reasonable request.

## Abbreviations

WHO: World Health Organization
